# Exploring Imagined Movement for Brain–Computer Interface Control: An fNIRS and EEG Review

**DOI:** 10.3390/brainsci15091013

**Published:** 2025-09-19

**Authors:** Robert Finnis, Adeel Mehmood, Henning Holle, Jamshed Iqbal

**Affiliations:** 1 School of Digital and Physical Sciences, Faculty of Science and Engineering, University of Hull, Hull HU6 7RX, UK; 2 School of Psychology and Social Work, Faculty of Health Sciences, University of Hull, Hull HU6 7RX, UK

**Keywords:** BCI, EEG, fNIRS, neuroimaging, motor imagery, neuroprosthetics, review

## Abstract

Brain–Computer Interfaces (BCIs) offer a non-invasive pathway for restoring motor function, particularly for individuals with limb loss. This review explored the effectiveness of Electroencephalography (EEG) and function Near-Infrared Spectroscopy (fNIRS) in decoding Motor Imagery (MI) movements for both offline and online BCI systems. EEG has been the dominant non-invasive neuroimaging modality due to its high temporal resolution and accessibility; however, it is limited by high susceptibility to electrical noise and motion artifacts, particularly in real-world settings. fNIRS offers improved robustness to electrical and motion noise, making it increasingly viable in prosthetic control tasks; however, it has an inherent physiological delay. The review categorizes experimental approaches based on modality, paradigm, and study type, highlighting the methods used for signal acquisition, feature extraction, and classification. Results show that while offline studies achieve higher classification accuracy due to fewer time constraints and richer data processing, recent advancements in machine learning—particularly deep learning—have improved the feasibility of online MI decoding. Hybrid EEG–fNIRS systems further enhance performance by combining the temporal precision of EEG with the spatial specificity of fNIRS. Overall, the review finds that predicting online imagined movement is feasible, though still less reliable than motor execution, and continued improvements in neuroimaging integration and classification methods are essential for real-world BCI applications. Broader dissemination of recent advancements in MI-based BCI research is expected to stimulate further interdisciplinary collaboration among roboticists, neuroscientists, and clinicians, accelerating progress toward practical and transformative neuroprosthetic technologies.

## 1. Introduction

With advancements in neuroimaging techniques, BCIs are becoming more accessible and affordable for a wider range of individuals [1,2]. BCIs allow for the control of external devices using brain signals, enabling individuals to interact with their environment in ways that were previously impossible. This technology has the potential to significantly improve the quality of life for those with mobility impairments or limb loss. The integration of BCIs with prosthetic limbs has been a significant focus of research, as it allows for more intuitive control and better functionality. By decoding brain signals, BCIs can translate the user’s intentions into actions performed by the prosthetic limb, providing a more natural and efficient way to interact with the world. One of the main challenges for BCI-based applications is the need for accurate and reliable signal processing techniques. The quality of the brain signals can be affected by various factors, including noise, artifacts, and individual differences in brain activity. Therefore, developing robust signal processing methods is crucial for improving the performance of BCI systems [3].

There have been two modalities chosen for this review: Electroencephalography (EEG) and functional Near-Infrared Spectroscopy (fNIRS). These modalities have been chosen due to their non-invasive nature, portability, and ability to provide real-time feedback, making them suitable for practical applications in BCI-based prosthetic control [1,2]. The modalities are also widely used in BCI research and have shown promise in decoding motor intentions from brain signals [4]. EEG measures electrical activity in the brain through electrodes placed on the scalp, while fNIRS measures changes in blood oxygenation levels in the brain using near-infrared light. Both modalities have their own advantages and limitations, which will be discussed in detail later in this review.

BCIs are controlled using cognitive or motor tasks, such as imagining movement or performing mental calculations. These tasks generate specific patterns of brain activity that can be detected and interpreted by the BCI system. Whilst motor imagery and motor execution tasks share similar components in the motor pathways [5], subjects can often struggle with motor imagery based approaches. It is often found that the time taken to complete motor imagery tasks is longer than that of motor execution tasks [6]. For the BCI-based control of a prosthetic limb, the user will typically perform a motor imagery task due to the loss of limb and resulting inability to execute the movement physically. This presents a unique challenge for BCI systems, as they must accurately decode the user’s intentions from imagined movements rather than actual physical actions [7].

Prostheses are used by amputees who have lost limbs due to an amputation from illness, accident, trauma or missing limb from birth; amputations cause a loss of motor control and sensory feedback for individuals, which can significantly impact their quality of life [8,9,10]. The integration of BCIs with prosthetic limbs has been a significant focus of research, as it allows for more intuitive control and better functionality. By decoding brain signals, BCIs can translate the user’s intentions into actions performed by the prosthetic limb, providing a more natural and efficient way to interact with the world [11,12,13]. Prosthetics used for upper limb amputees are typically controlled through bio-signals, which are electrical signals generated by muscle contractions. However, these control systems can limit a users control and dexterity. In order to improve the functionality of prosthetic limbs, researchers have been exploring the use of BCIs to provide more direct and intuitive control.

To develop a robust BCI system, a user will learn a new skill to control the system. BCI skills are similar to natural learn skills as they require practice and adaptation to achieve proficiency. But even the simplest skill can take many hours to learn, and the speed at which a user can learn a new skill is dependent on the individual [4,14]. With training and practice, users can learn to improve the dexterity and control of a BCI device. In preliminary studies, users have classified discrete movement classes but noted that there is a lack of continuous kinematic reconstruction [15,16].

Within BCI research, experiments are commonly classified as either offline or online. Offline experiments involve collecting brain signal data and analyzing it after the session has ended, allowing researchers to develop and validate signal processing and classification algorithms without the constraints of real-time processing. In contrast, online experiments require the system to process brain signals and provide feedback or control external devices in real time, closely simulating practical, real-world applications. While offline experiments are essential for algorithm development and benchmarking, online experiments are crucial for evaluating the usability, robustness, and responsiveness of BCI systems in dynamic environments. Both approaches play a vital role in advancing BCI technology for prosthetic control.

This review aims to explore the current state of EEG and fNIRS-based BCI technology, focusing on how well the modalities can be used to predict imagined movement for offline and online applications. The review will also discuss the challenges and limitations of these modalities, as well as the potential for future research and development in this area.

## 2. Methods

**Figure 1:**
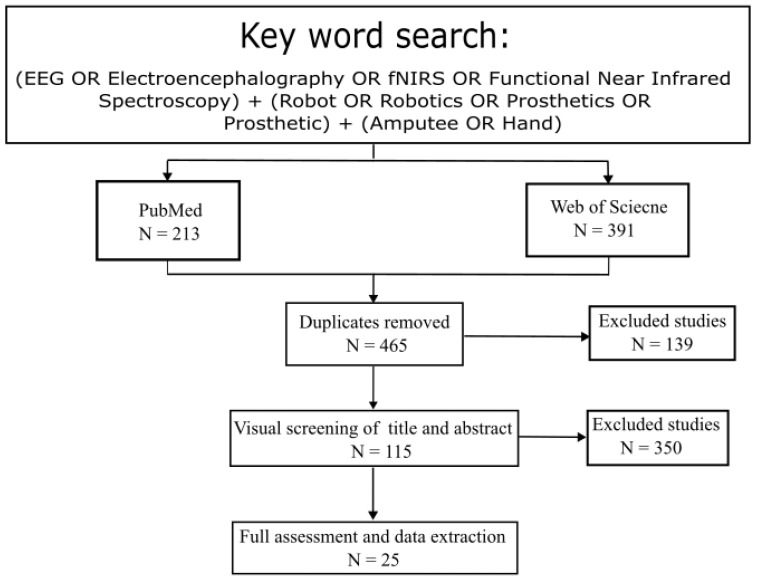
Overview of literature search for review.

In this study, a two-stage literature search was performed which identified the relevant investigations. The first approach was an online search targeting two popular databases: PubMed, Web of Science. These consisted of peer-reviewed articles from January 2017 to September 2024 with the following keywords: (EEG OR Electroencephalography OR fNIRS OR Functional Near Infrared Spectroscopy) + (Robot OR Robotics OR Prosthetics OR Prosthetic) + (Amputee OR Hand). Additional literature was identified through the reference list in selected studies or from relevant review papers. The aim of the two-stage literature search was to ensure that the identified studies were as comprehensive as possible. As shown in Figure 1, the search returned 604 studies with 213 from PubMed and 391 from Web of Science. After removing 139 duplicates and 350 studies that did not related to this review (i.e., review studies, stroke rehabilitation, autonomous navigation, lacking fNIRS or EEG), 105 articles remained.

**Figure 2:**
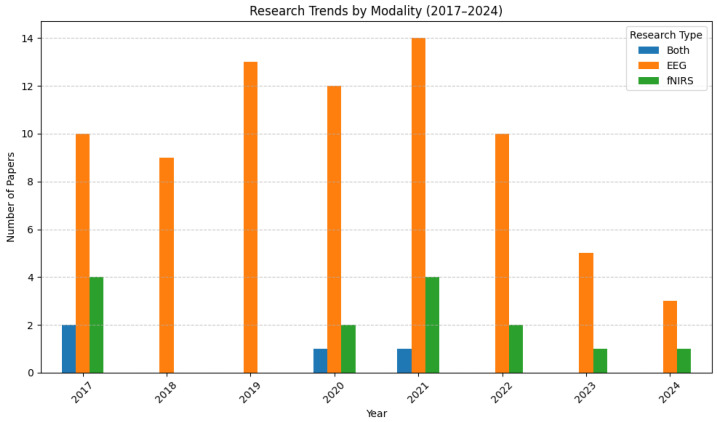
Papers published, grouped by neuroimaging modality.

Figure 2 presents the distribution of studies based on the neuroimaging modality used—EEG, fNIRS, or a combination of both. EEG has remained the dominant technique across all years, while fNIRS-only studies have consistently been in the minority. However, hybrid EEG–fNIRS studies, although limited in number, highlight a growing interest in leveraging the complementary strengths of both modalities. The continued preference for EEG may reflect its maturity and availability, but the increased proportion of studies using fNIRS suggests rising recognition of its value, particularly in hybrid systems aimed at enhancing classification performance and reducing signal noise.

## 3. Background

For this review, the applications considered are those that use a non-invasive technology that can acquire brain activity for a BCI device. BCI devices allow for the control of an external device by interpreting signals that have been extracted from the central nervous system [17,18,19]. There are two categories of BCI devices: invasive and non-invasive. One of the challenges of non-invasive neuroimaging techniques is the inaccessibility of measuring deep neural activity in the brain reliability; as Section 3.3 will discuss, it is difficult to control BCI devices through imagined movements without numerous hours in training a BCI. This can be compounded by BCI applications for amputees where a limb amputation causes immense emotional trauma and stress on the brain, consequently the loss of a limb re-wires the neural pathways in the brain [20,21].

### 3.1. EEG

EEG is a widely used neuroimaging technique that measures electrical activity in the brain through electrodes placed on the scalp [22,23,24,25]. EEG is able to record the brain’s electrical potentials by deriving the activation of the cerebral cortex; which could be used to spontaneous or evoked brain activity [26]. The signals recorded are a time series; this time series is acquired through non-invasive electrodes (often referred to as channels) which are places on the scalp of a subject [27,28,29]. For high-level accuracy, the electrode positioning follows standard placement systems such as the 10–20 or 10–10 Internation System. These systems define the distance between the electrodes using anatomical landmarks as references such as the distance from the nasion to the inion, and the distance between the left and right preauricular points [28,30]. The systems are such that the electrodes are placed according to the brain’s anatomical structure; however, the neural activations of the brain are not always recorded uniformly and this can lead to imprecise recordings [31].

EEG is not a one-size-fits-all solution for recording brain activity as it has some limitations. EEG signals are easily affected by noise and therefore usually have low signal-to-noise ratio [32,33]. This is a problem when using EEG signals in a real-world application where it is not possible to block signals such as bluetooth, Wi-Fi, and cellular data [34]. Furthermore, the signal quality in EEG recordings is highly dependent on the electrode-scalp interface. To optimise the conductivity and reduce impedance it is often necessary to cleanse the scalp and apply conductive materials such as NeuroPrep gel or Ten20 paste [27]. While this preparation enhances the signal fidelity, it can residual substances on the scalp and hair, necessitating in post-session cleaning.

### 3.2. fNIRS

fNIRS is a non-invasive neuroimaging technique that uses near-infrared light to measure the changes in the oxygenated and de-oxygenated hemoglobin (HbO/HbR) levels in a targeted brain region, which is a consequence of neurovascular coupling. When a specific brain region becomes active due to cognitive or motor processes, there is an associated increase in the metabolic demand for oxygen and glucose [4,35]. Initially, this results in a transient increase in local oxygen consumption, leading to a slight elevation in HbR concentration. However, to compensate for this increased metabolic demand, the body initiates a rapid and localized increase in cerebral blood flow, which is measured by the fNIRS optodes [9,36]. A source emits near-infrared light into the scalp, while detectors positioned a few centimeters away pick up the reflected light after it has passed through the cortical tissue. When the infrared light is shone through the brain, it is scatted due to the change in direction of the light as it passes through biological tissue. The reflected light interacts differently with oxygenated and deoxygenated blood, in which the amount of light absorbed enables the system to track the blood oxygenation. Consequently, this allowed for an indirect measure of brain activity from neural processes through the changes in blood flow and oxygenation in response to neural demand.

As fNIRS measures the hemodynamic response in the brain, it is not susceptible to movement artifacts, which are so prominent in other neuroimaging techniques such as eye blinks. However, it is common for fNIRS data to be visually inspected to correct the data in case of any identified artifacts [37]. Further to this, fNIRS is better suited to real-world environments [38], as electrical signals do not affect the data as the reflected light in the brain is recorded via flexible fiber optic cables, which allow neuroimaging experiments to be conducted on participants while performing tasks such as standing or walking [9,39].

In fNIRS, the reflected light does not penetrate the brain very deep. fNIRS can only scan the cortical tissue and the maximum depth it can scan is 2–3 cm with it typically only reaching a depth of 1.5 cm [40,41].

### 3.3. Motor Imagery

Motor Imagery (MI) is the mental stimulation of body movements. This works by a user consciously accessing a motor representation; they will show intention of making a movement [35,42]. There have been numerous studies that have implemented MI as a means to control external BCI prosthetics with common movement classes of open hand, close hand, wrist flexion, and wrist extension [16,43,44,45].

MI-based BCIs are a good non-invasive alternative for applications such as prosthetics due to the current myoelectric methods relying on residual muscles in the forearm, which can make it difficult to identify the precise action that is intended by the user, especially after an amputation where nerves have been damaged and repaired [7,10]. For fine motor control in the wrist and finger movements, MI is more challenging to identify and reproduce, as the region of interest in the brain is a significantly smaller area of activation in the sensorimotor cortex. This is further compounded by the lower spatial resolution that non-invasive neuroimaging techniques provide [45].

EEG signals from the primary sensorimotor cortex contain oscillations known as sensorimotor rhythms. These rhythms are predominantly observed in the γ and β bands. The rhythms undergo event-related amplitude modulations which are classified as event-related synchronization and synchronization. Event-related synchronization refers to a decrease in the rhythmic amplitudes and is commonly associated with cortical activation whereas event-related synchronization represents an increase in rhythmic amplitude, generally corresponding to cortical idling or inhibition [14]. The event refers to any cognitive, motor or sensory signal that is strong enough to register as a neural response. These are not the only way to identify movement in EEG signals, evoked potentials are characterized by stereotyped, time-locked positive and negative deflections in EEG voltage. Some EEG paradigms like P300 or steady-state visual evoked potentials do not rely on spontaneous modulations of sensorimotor rhythms and are stable oscillations in voltage that are elicited by rapid repetitive stimulation such as strobe light, LED [46].

MI-based BCIs can be slow and suffer from low accuracy due to a significant amount of training that needs to be done by a user. However, the MI ability is subjective due to the uniqueness of the individuals that take part in MI studies. This is identified in MI sessions and can impact on the amount of training that an individual needs [47]. MI is often used in offline experiments due to the method requiring a lot of computational power to classify the movements [24,48].

### 3.4. Motor Execution

Whilst MI is used for BCI-based prosthetics; when BCIs are in the initial phase of experimentation or being used for offline experiments as will be discussed in Section 3.5, healthy individuals are often recruited for studies [49,50]. The purpose of this is to set a baseline for future experiments that conduct studies on BCI applications. One BCI application is for prosthetic limb control; here, motor execution cannot be used for online use but is used to determine the success of offline machine learning and deep learning techniques [22,51]. Motor execution is frequently used as the performance benchmark in review studies evaluating the accuracy of MI-based BCIs. Motor execution provides a reliable platform for assessing the effectiveness of the machine learning and deep learning techniques [32,52,53].

Motor execution-based BCIs perform better than MI-based BCIs due to a clear motor cortex activation; this results in higher classification accuracy and participants find it easier to reproduce the movement compared to MI where they can struggle to reliably repeat the motion [32,34,35].

### 3.5. Offline/Online Experiments

There are two types of experimental research: amongst data-driven research, there is either online experiments or offline experiments [3]. Online experiments are conducted in real-time environments where user behaviour is continuously monitored and observed. Numerous research studies have found that online experiments struggle with the performance of classification methods [3,24,43,48]. It can be difficult to achieve high classification accuracy and low latency in these experiments, but it is possible to predict motions [9,16,38,54].

Offline experiments are generally more computationally expensive and often yield more reliable and accurate classifications [45,55,56]. The advantage of offline experimentation is that machine learning algorithms can be applied to the data gathered to help improve the classification accuracy of predicted movements [45,52,56,57].

**Figure 3:**
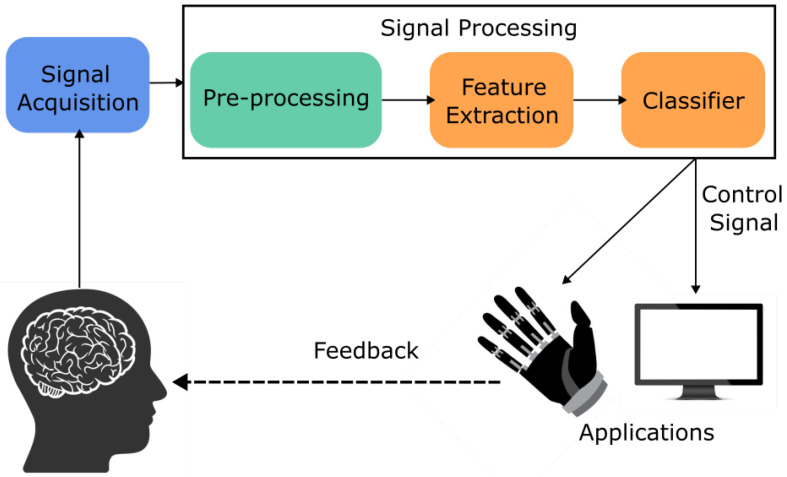
BCI pipeline for EEG-based online experiment.

**Figure 4:**
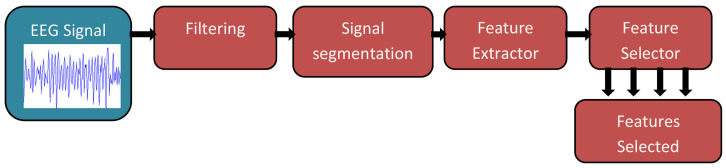
BCI pipeline for EEG-based offline experiment [61].

For a BCI-device to be effective, they will typically have two phases [33]; the initial phase will be offline experimentation on healthy individuals where non-invasive neuroimaging techniques will be used to gather data and then classify movements [28,39,58]. The offline phase can also be a chance to fine-tune the deep learning model that will be utilised in the second phase [33]. Following an online phase is generally an online phase that would include healthy individuals or individuals with disabilities that fit the criteria for that BCI application, such as amputees for prosthetic prediction control [9,59,60]. Figure 3 and Figure 4 show an overview of an online and offline pipeline used for experiments.

Both offline and online experiment complement each other. Whilst offline experiments provide accuracy and robustness [28,55], online experiments ensure real-time applicability [53]. Recognising their respective trade-offs is essential when design effective BCI systems.

### 3.6. Summary of Modalities and Motor Imagery Viability

This section established the foundational concepts and challenges involved in using non-invasive neuroimaging techniques—particularly EEG and fNIRS—for brain–computer interface (BCI) systems. It discussed the physiological and technical constraints inherent in interpreting brain signals, especially from individuals with amputations, where neural plasticity and emotional trauma can rewire cortical pathways. While EEG offers high temporal resolution and is sensitive to changes in sensorimotor rhythms during Motor Imagery (MI), it is highly susceptible to noise. Conversely, fNIRS offers better resilience to movement artifacts and environmental interference but suffers from limited depth and delayed hemodynamic response. MI, which is critical for amputees who cannot physically execute movement, poses particular challenges due to individual variability, low spatial resolution, and the significant training required. Nevertheless, both modalities show potential for decoding MI, especially in offline contexts where computational demands can be met. Overall, while MI prediction is viable, it remains less accurate and slower than motor execution tasks due to these physiological and technical limitations.

## 4. Overview of BCI Experiments

This section is aimed to review recent experimental for EEG, fNIRS, and hybrid EEG-fNIRS modalities with a primary focus on MI with motor execution and how well-imagined movement can be predicted. It examines the differences in processing the raw signal data for feature extraction and classifiers, touching on both offline and online experiments for traditional methods and newer deep learning and machine learning approaches to predict user intention.

### 4.1. EEG-Based BCI Experiments

EEG has shown promise as a modality for use in BCI-based applications [23,24,54,62]. However, the main issue associated with EEG signals in BCI-based applications is the induced movement artifacts that are observed [50,57,63]. These can be intentional movements or spontaneous movements such as eye blinks or muscle twitches that impact the quality of the EEG signals [64,65,66]. Unintentional responses or artifacts can be removed through visual inspection and, in some cases, a channel or trial will be removed entirely from the data [67].

Table 1 presents an overview of EEG-BCI studies for determining how well-imagined movement can be predicted. The commonly used brain regions for offline studies were the sensorimotor cortex which includes the primary motor cortex and somatosensory cortical area [32,55,68]. Some studies that use commercial equipment such as the Emotiv Epox [11,53,69] do not have the same adaptability to choose which channels they use on the 10–20 international system. They are confined to use 14 specific channels with the headset getting minimal coverage over the entire brain.

Depending on whether the study is online or offline, analyzing EEG signals can use the same techniques; the most common method to extract the features from the EEG signals is Movement-related Cortical Potentials, Independent Component Analysis (ICA) and Linear Discriminant Analysis (LDA) [28,32,36,70]. ICA is a method that separates signals into additive subcomponents [11,71], transforming EEG signals into a format so that they can be generalized. Other processes such as LDA can be applied to reduce the dimensionality of the signals. LDA is a linear classifier that projects signals/data onto a lower-dimensional space; this helps identify the most relevant features that can contribute to a targeted movement [11,72]. Thus, LDA reduces the computational complexity and makes a more suitable implementation for online applications [23]. Offline stuides are generally used to improve the classification accuracy and will often compared multiple classification methods together to find the optimum method for a future study [28,55,70,73]. Offline studies are crucial for validating the effectiveness of different feature extraction techniques and selecting the appropriate classifiers for experimentation. SVM is a preferred choice for multi-class classifications where time is not a factor and offline experiments as it is computationally expensive [32,45,73].

Online BCI studies validate the performance of models for real-time constraints such as controlling prosthetic limbs. These systems must be able to process the raw EEG signals, extract features, and classify the movements with minimal latency and high tolerance and robustness [55,59,65]. For online experiments, LDA is a common classifier used. This is due to the low computational complexity where the accuracy of the classifier and speed are vital [23]. However it is possible to implement similar dimensional reducing methods such as principle component analysis and SVM to achieve similar results [53].

**Table 1:** EEG–BCI studies for motor imagery and motor execution movement prediction.

Study	Paradigm	Brain Area	Features	Classifier	Type of Study
Kansal et al. [55]	Motor Execution	Sensorimotor Cortex	Time-domain, Min–Max scaled	Genetic Algorithm optimized Long Short-Term Memory	Offline
Mikson et al. [69]	Motor Execution + Facial	Frontal/Temporal	FFT, ICA	Thresholding	Online
Staffa et al. [52]	Motor Imagery	FC, C, Cz channels	Wavelet-based decomposition	WiSARD (Weightless Neural Network)	Offline training; Online execution
Carnio-Escobar et al. [23]	Motor Imagery	F3-P4 (16 channels)	CSP	LDA	Online
Xu et al. [28]	Motor Execution	Frontal and Parietal	MRCP	LDA	Offline
Xu et al. [70]	Motor Execution	Frontal and Parietal	MRCP	LDA	Offline
Cho et al. [32]	Motor Execution + Motor Imagery	Sensorimotor Cortex	LDA	SVM	Offline
Faiz & Al-Hamadani [53]	Motor Execution + Motor Imagery	Frontal, Parietal, and Temporal lobes	Autoregressive + CSP	PCA + SVM	Online
Gayathri et al. [11]	Motor Imagery	Fontal, Parietal and Temporal lobes	ICA	LDA	Offline
Shantala & Rashmi [73]	Motor Imagery	Frontal, Parietal and Temporal lobes	Wavelet Transform	LDA, SVM, kNN	Offline
Alazrai et al. [45]	Motor Imagery	Sensorimotor Cortex	Convolutional Neural Network	Convolutional Neural Network	Offline

Advancements in machine learning and deep learning algorithms have reduced the quantity of manual feature extraction and classification implementations. MI-based studies are a prime focus for deep learning and offline studies where the algorithms can produce high accuracy classifications and reliability [51,54,63,66]. Common methods implemented are convolutional neural networks and long short-term memory algorithms for feature extraction and classification [45,55,74,75]. Alazrai et al. [45] found that a custom neural network improved the accuracy for able-bodied and amputees by 14.5% and 11.2%, respectively. The classification increase was using 18 able-bodied and 4 amputees. This is in comparison to the implementation by Kansal et al. [55] where the optimized classification accuracy improved on average by 19.55%. Whilst both where trained in offline experiments, the models can be applied to real-time situations directly as both models only took up approximately 20% of the sliding window used in EEG classifications [45,55]. Advancements have been made in processing multiple EEG frequency bands and applying recurrent neural networks to them [75]. They found that long short-term memory models that were trained on segregated frequency channels were able to learn temporal correlations across rhythms which can reduce noise sensitivity. Long short-term memory models combined with recurrent neural networks are able to be trained to anticipate upcoming human motor movements [76]. These anticipated movements can improve the safety of BCI devices.

Transitioning from offline to online BCI systems requires consideration for the computational latency, classifier accuracy, and robustness to real-world noise [34,77]. The classifiers used must perform reliably on signals that are being observed in non-clinical environments and to the subject over time [22,63]. There have been studies that have been able to demonstrate the effective integration of offline-trained models in robust online BCI systems [23,52]. These models have been trained on offline datasets [78] or experiments [48] and the parameters of the algorithms are uploaded onto the BCI for real-time decision-making. Online BCI applications require the need to have an adaptable neural network, as the classifiers can be subjected to unexpected data such as unintentional movement. For this, SVM is an ideal classifier but due to the computational load, lightweight classifiers such as LDA are utilized; weightless neural networks also show promise here and these networks can be used to find the relationship in high dimensional spaces without processing the signals through ICA or LDA [52,62,63].

### 4.2. fNIRS-Based BCI Experiments

As shown in Table 2, fNIRS research employs both MI and motor execution paradigms, with motor execution studies generally outnumbering MI. This trend aligns with the stronger and more consistent hemodynamic responses elicited during actual movements, facilitating signal decoding [79,80]. However, MI paradigms remain critical for BCI applications targeting users unable to perform physical movements, such as amputees or patients with severe motor impairments [4,9,81].

fNIRS has shown promise over recent years as a potential for portable BCI applications [9,35,79]. However, fNIRS has an inherent physiological delay of approximately 2 s, which poses challenges for real-time control [79]. fNIRS can be used for both online and offline experiments [4,79,81], with online experiments commonly being integrated into hybrid BCI-based research [17,82,83] as discussed in Section 4.3.

Offline fNIRS studies allow for researchers to not be limited by the physiological delays that occur when measuring the changes in HbO and HbR levels in the blood. It also allows for analysis of the pipeline notably the classifiers under controlled conditions [35,81]. The most common feature extraction method of fNIRS is to use the signal mean, signal slope, signal variance, signal skewness, kurtosis, and signal peak [4,35,37]. Nazeer et al. [81] proposed an alternative way to identify the active channels from MI data that are used for classification, the z-score-based method improved the accuracy of the LDA classifier on average by 10% in comparison to standard methods that identify active regions of the brain by Δhemodynamic response. Four daily-life arm movements (lifting, putting down, pulling back, and pushing forward) can also be identified using fNIRS and achieve high offline classification accuracies, with Random Forest reaching 94.4% and SVM achieving 84.4% [84] for multi-class recognition.

Online BCI systems using the fNIRS modality often struggle with the slower hemodynamic response but benefit from the robustness to motion artifacts [4,9,38]. Similarly to EEG, LDA is a popular classifier to use for online studies due to SVM requiring more resources and inducing more latency in a modality [79].

fNIRS has also been applied to predict motion movements. This has shown that there is a stronger activation in the inferior and superior parietal lobes under equal time structure conditions to predict arrival time of moving objects [85].

fNIRS-based systems will typically use HbO and HbR signals as the inputs for machine learning models, Ortega & Faisal [80] explores the application of deep learning for offline fNIRS studies, focusing on decoding bimanual grip force from multimodal fNIRS and EEG signals. The study introduces a novel deep-learning architecture, which incorporates residual layers and self-attention mechanisms to enhance the fusion of EEG and fNIRS signals. The fNIRS data is processed using the modified Beer–Lambert law. The deep-learning models outperform traditional linear methods in reconstructing force trajectories, detecting force generation, and disentangling hand-specific activities. This highlights the advantages of deep learning in capturing non-linear modulations of brain signals and improving the fusion of multimodal data, offering significant potential for advancing offline fNIRS studies in motor control and brain–machine interface applications [80,86].

### 4.3. Hybrid BCI-Based EEG & fNIRS

Notably, a majority of recent EEG-fNIRS hybrid BCI studies have employed motor execution paradigms rather than purely MI tasks, as reflected in Table 3. This predominance is likely due to motor execution providing clearer neurophysiological signals and more robust hemodynamic responses, facilitating initial algorithm development and system validation [17,80]. However, motor execution requires actual movement, which is not feasible for many amputees or severely impaired users.

In contrast, MI-based paradigms, though less represented in the current literature, remain critical for applications where voluntary movement is limited or absent. MI poses greater challenges due to weaker and more variable cortical activations, resulting in lower classification accuracies and increased training demands [89]. Therefore, ongoing research aims to improve MI decoding through advanced signal processing and multimodal fusion with fNIRS to compensate for its inherent signal limitations [22,87]. Bridging this gap is essential for developing clinically viable hybrid BCIs tailored to users with motor disabilities.

Hybrid BCIs utilizing EEG and fNIRS modalities have emerged as a promising approach in the development of neuroprosthetics and assistive technologies for rehabilitation [17,36,80]. Among the various signal combinations, EEG and fNIRS are particularly effective for decoding MI, which allows users to mentally simulate movements—such as grasping or reaching—without physical execution. This capability is crucial for individuals with upper-limb amputations, especially where muscle signals are not viable due to tissue damage or limb absence [7,90,91,92].

EEG provides high temporal resolution, capturing fast neural activity during MI, while fNIRS measures hemodynamic responses related to cortical activation. This complementary relationship enhances the reliability and accuracy of movement prediction. However, practical implementation of hybrid systems remains complex due to increased computational demands and sensor setup requirements [17,93,94].

To address these challenges, channel selection techniques such as Sequential Backward Floating Search have been employed to reduce EEG data dimensionality while retaining MI-relevant features [22]. While traditional MI-based EEG systems may struggle with low accuracy and extensive training requirements [89], the integration of fNIRS can compensate by providing spatially localized information that reinforces EEG signals during MI tasks [4,89].

Several studies have demonstrated that hybrid BCI devices enhances classification performance in multi-degree-of-freedom prosthetic control [36,80,94], enabling the differentiation of imagined reaching, grasping, or lifting actions. Furthermore, strategic electrode placements—such as embedding EEG electrodes between fNIRS optodes—can improve cortical coverage without signal interference [80].

Recent deep learning approaches, including convolutional neural networks, have been applied to extract event-related synchronization/desynchronization features from EEG-fNIRS data, offering improved classification of MI-related tasks [87]. These hybrid techniques improve long-term stability and accuracy, making them viable for real-world applications in rehabilitation and assistive technologies [26,88,95,96].

### 4.4. Summary of Experimental Findings and Predictive Performance

This section reviewed numerous BCI experiments using EEG, fNIRS, and hybrid EEG-fNIRS systems to predict motor imagery and execution tasks. Offline EEG studies demonstrate strong performance, particularly when using advanced machine learning models like CNNs and LSTMs, with several studies reporting classification improvements of up to 20%. These models, though trained offline, can be adapted for online use due to their efficiency in feature extraction and classification. Online EEG experiments, while more challenging due to real-time constraints and artifacts, show growing reliability, especially when lightweight classifiers like LDA are employed. fNIRS-based studies also validate the capability to predict MI, though generally with lower temporal resolution and a physiological delay. However, fNIRS offers improved motion artifact robustness and remains promising, particularly in hybrid systems. Hybrid EEG-fNIRS systems leverage the strengths of both modalities, enhancing MI classification accuracy by combining EEG’s fast response with fNIRS’s spatial precision. In conclusion, the prediction of online imagined movement is possible and improving, but accuracy and speed still lag behind motor execution. Continued integration of modalities and algorithmic advancements are narrowing this gap.

## 5. Conclusions

This systematic review has explored the comparative strengths and limitations of EEG and fNIRS in BCI systems for prosthetic limb control. While EEG has long been the cornerstone of non-invasive neural interfaces due to its high temporal resolution and affordability, it remains limited by spatial resolution, susceptibility to motion artifacts, and signal noise in real-world environments. In contrast, fNIRS offers a compelling alternative, with its robustness to motion artifacts, improved spatial resolution, and enhanced usability in mobile and less controlled settings.

Despite a lower temporal resolution due to the hemodynamic delay, fNIRS demonstrates significant potential in decoding motor intention, particularly in tasks involving gross and fine motor control. Recent advances in real-time processing, lightweight hardware, and hybrid systems further demonstrate its viability. Experimental results consistently show that fNIRS-based systems can achieve reliable classification accuracies for both motor execution and imagery tasks, especially when combined with efficient signal processing and machine learning techniques.

fNIRS is increasingly proving its value not only as a stand-alone modality but especially as a core component in hybrid BCI systems. When combined with EEG, fNIRS compensates for EEG’s shortcomings, particularly its low spatial resolution and vulnerability to noise and using the hemodynamic response, it can enhance signal robustness and classification accuracy. Hybrid systems leveraging both EEG and fNIRS benefit from the high temporal resolution of EEG and the spatial precision and motion artifact resilience of fNIRS, creating a more comprehensive and adaptive neural interface. This integration improves the detection of complex motor intentions, reduces cognitive load, and enables more precise control of prosthetic devices. Therefore, fNIRS is not only an alternative but also a well-suited modality to complement EEG in hybrid BCIs, offering a more dependable and scalable path forward for neuroprosthetic applications and assistive technologies.

Notably, analysis of publication trends between 2017 and 2024 reveals a peak in research output around 2021, with a marked decline in the following years. While this may not directly coincide with the peak of the COVID-19 pandemic, the lag in publication timelines suggests that restrictions on in-person experimentation during the pandemic likely delayed data collection and disrupted research involving human participants. This disruption is reflected in the drop in published studies post 2021, particularly those involving novel data acquisition or user trials which are key aspects of BCI research.

Future research should focus on the advancements in hybrid BCI framework to optimised the strength that resonate from EEG and fNIRS. Further, it should also address the challenges of portability, long-term usability and real-time performance. Expanding research into clinical trials with diverse participant groups and not just healthy individuals will be critical to validate these systems in real-world environments and accelerate the translation into practical neuroprosthetic applications.

## Figures and Tables

**Table 2:** fNIRS-BCI studies for motor imagery and motor execution movement prediction.

Study	Paradigm	Brain Area	Features	Classifier	Type of Study
Batula et al. [4]	Motor Imagery	Motor and Supplementary Motor Cortex	Mean, Median, Max, Slope	LDA	Online
Lee et al. [79]	Motor Execution	Motor Cortex	Mean and Slope	LDA, SVM	Online
Nazeer et al. [81]	Motor Imagery	Frontal, Motor, and Visual Cortex	z-score channel selection	LDA	Offline
Sattar et al. [9]	Motor Imagery	Prefrontal Cortex	Signal Peak and Mean, Signal Dip	LDA	Offline
Asgher et al. [35]	Motor Execution	Prefrontal Cortex	Signal Mean, Slope, Variance, Skewness, Kurtosis, Peak	SVM	Offline
Ortega & Faisal. [80]	Motor Execution	Sensorimotor Cortex	Modified Beer–Lambert law	LSTM, CNN	Offline

**Table 3:** EEG/fNIRS-BCI studies using for movement prediction.

Study	Paradigm	Brain Area	Features	Classifier	Type of Study
Tang et al. [22]	Motor Imagery	Motor Cortex	Channel selection, ERD/ERS	SVM	Offline
Kim et al. [87]	Motor Execution	Sensorimotor Cortex	ERD/ERS + 2D CNN input	CNN	Offline
G. Zhu et al. [17]	Motor Execution	Frontal, Motor Areas	Temporal + HbO/HbR features	LDA	Online
Ortega & Faissal [80]	Motor Execution	Sensorimotor Cortex	Modified Beer–Lambert law	LSTM, CNN	Offline
Bandara et al. [36]	Motor Execution	Frontal + Parietal	EEG/fNIRS fusion features	Naive Bayes	Offline
Mavridis et al. [88]	Motor Execution	Frontal/Occipital	EEG error signals	Regression Model	Online
